# Supplementary material for: Gene Losses and Plastome Degradation in the Hemiparasitic Species Plicosepalus acaciae and Plicosepalus curviflorus: Comparative Analyses and Phylogenetic Relationships among Santalales Members
Source: Plants (Basel). 2022 Jul 18;11(14):1869. doi: 10.3390/plants11141869 (PMC9317152; doi:10.3390/plants11141869)
Supplement: Supplementary file 1 [file plants-11-01869-s001.zip › plants-1717410-supplementary.pdf]

Table S1: Genes present in the plastome of *P. acaciae*.

Category	Group of genes	Name of genes
RNA genes	Ribosomal RNA genes (rRNA)	rrn4.5S[a], rrn16S[a], rrn5S[a], rrn23S[a]
	Transfer RNA genes (tRNA)	trnR-UCU, trnC-GCA, trnT-GGU, trnG-GCC, trnS-GGA, trnF-GAA, trnM-CAU[a], trnV-GAC[a], trnR-ACG[a], trnL-UAG, trnN-GUU[a], trnL-CAA[a], trnH-GUG[a], trnP-UGGa, trnW-CCA[a], trnT-UGU, trnS-UGA, trnE-UUC, trnY-GUA, trnD-GUC, trnS-GCU, trnQ-UUG, trnA-UGC, trnI-CAU, trnL-UAA[+], trnI-GAU[+]
Ribosomal proteins	Small subunit of ribosome	rps11, rps12a[+], rps14, rps15, rps18, rps19, rps2, rps3, rps4, rps7[a], rps8, rps18
Transcription	Large subunit of ribosome	rpl14, rpl16, rpl2[+], rpl20, rpl22, rpl23[a], rpl36
	DNA dependent RNA polymerase	rpoA, rpoB, rpoC2, rpoC1[+]
Protein genes	Photosystem I	psaA, psaB, psaC, psaI, psaJ
	Photosystem II	psbA, psbB, psbC ,psbD, psbE, psbF, psbH, psbI, psbJ, psbK, psbM, psbT, psbZ, psbN
	Subunit of cytochrome	petA, petB[+], petD, petG, petL, petN
	Subunit of synthase	atpA, atpB, atpE, atpF[+], atpH, atpI
	Large subunit of RUBISCO	rbcL
	Chloroplast envelope membrane protein	cemA
Other genes	Maturase	matK
	Subunit acetyl-coA carboxylase	accD
	C-type cytochrome synthesis	ccsA
	Hypothetical proteins	ycf2, ycf15[a],ycf3[++],ycf4
	Component of TIC complex	ycf1[a]
	Protease	clpP[++]

+ Gene with one intron, [++] Gene with two intron and [a] Gene with copies

Table S2: Genes present in the plastome of *P. curviflorus*.

Category	Group of genes	Name of genes
RNA genes	Ribosomal RNA genes (rRNA)	rrn4.5[a], rrn16[a], rrn5[a], rrn23[a]
	Transfer RNA genes (tRNA)	trnR-UCU, trnC-GCA, trnT-GGU, trnG-GCC, trnS-GGA, trnF-GAA, trnM-CAU[a], trnV-GAC[a], trnR-ACG[a], trnL-UAG, trnN-GUU[a], trnL-CAA[a], trnH-GUG, trnP-UGG, trnW-CCA, trnT-UGU, trnS-UGA, trnE-UUC, trnY-GUA, trnD-GUC,trnS-GCU,trnQ-UUG, trnL-UAA, trnI-CAU[a], trnA-UGC[a]
Ribosomal proteins	Small subunit of ribosome	rps11, rps12[++,a], rps14, rps15, rps18, rps19, rps2, rps3, rps4, rps7[a], rps8
Transcription	Large subunit of ribosome	rpl14, rpl16[+], rpl2[+], rpl20, rpl22, rpl23[a], rpl36
	DNA dependent RNA polymerase	rpoA, rpoB, rpoC1[+], rpoC2
Protein genes	Photosystem I	psaA, psaB, psaC, psaI, psaJ, ycf3[++], ycf4
	Photosystem II	psbA, psbB, psbC ,psbD, psbE, psbF, psbH, psbI, psbJ, psbK,psbN, psbM, psbT, psbZ
	Subunit of cytochrome	petA, petB[+], petG, petL, petN
	Subunit of synthase	atpA, atpB, atpE, atpF[+], atpH, atpI
	Large subunit of RUBISCO	rbcL
	ATP dependent protease subunit P	clpP1[++]
	Chloroplast envelope membrane protein	cemA
Other genes	Maturase	matK
	Subunit acetyl-coA carboxylase	accD
	C-type cytochrome synthesis	ccsA
	Hypothetical proteins	ycf2[a],ycf3[++],ycf4,ycf15[a]
	Component of TIC complex	ycf1

+ Gene with one intron, [++] Gene with two intron and [a] Gene with copies

Table S3: Codon-anticodon recognition patterns and codon usage in the *P. acacia* chloroplast genome.

Codon	Amino acid	RSCU	tRNA	Codon	Amino acid	RSCU	tRNA
UUU	Phe	0.76	trnF-GAA	UAU	Tyr	0.55	trnY-GUA
UUC	Phe	1.24		UAC	Tyr	1.45	
UUA	Leu	1.41	trnL-UAA	UAA	Stop	0	
UUG	Leu	1.24	trnL-CAA	UAG	Stop	0	
CUU	Leu	0.62	trnL-UAG	CAU	His	2	trnH-GUG
CUC	Leu	1.06		CAC	His	0	
CUA	Leu	0.79		CAA	Gln	0.81	trnQ-UUG
CUG	Leu	0.88		CAG	Gln	1.19	
AUU	Ile	0.91		AAU	Asn	1.47	trnN-GUU
AUC	Ile	1.18		AAC	Asn	0.53	
AUA	Ile	0.91	trnI-CAU	AAA	Lys	1.11	
AUG	Met	1	trnM-CAU	AAG	Lys	0.89	
GUU	Val	1.21	trnV-GAC	GAU	Asp	1.26	trnD-GUC
GUC	Val	0.89		GAC	Asp	0.74	
GUA	Val	1		GAA	Glu	0.5	trnE-UUC
GUG	Val	0.89		GAG	Glu	1.5	
UCU	Ser	0.68	trnS-GGA	UGU	Cys	1.57	trnC-GCA
UCC	Ser	1.91		UGC	Cys	0.43	
UCA	Ser	0.82		UGA	Stop	0	
UCG	Ser	1.02	trnS-UGA	UGG	Trp	1	trnW-CCA
CCU	Pro	1.33	trnP-UGG	CGU	Arg	0.97	trnR-ACG
CCC	Pro	1.33		CGC	Arg	1.24	trnR-UCU
CCA	Pro	0.72		CGA	Arg	0.79	
CCG	Pro	0.62		CGG	Arg	1.15	
ACU	Thr	1.58		AGA	Arg	0.53	
ACC	Thr	0.97		AGG	Arg	1.32	
ACA	Thr	0.61	trnT-GGU	AGU	Ser	0.55	trnS-GCU
ACG	Thr	0.85	trnT-UGU	AGC	Ser	1.02	
GCU	Ala	1.07	trnA-UGC	GGU	Gly	1	
GCC	Ala	1.07		GGC	Gly	1	trnG-GCC
GCA	Ala	0.67		GGA	Gly	0.56	
GCG	Ala	1.2		GGG	Gly	1.44	

Table S4: Codon-anticodon recognition patterns and codon usage in the *P. curviflorus* chloroplast genome.

Codon	Amino acid	RSCU	tRNA	Codon	Amino acid	RSCU	tRNA
UUU	Phe	0.62	trnF-GAA	UAU	Tyr	1	trnY-GUA
UUC	Phe	1.38		UAC	Tyr	1	
UUA	Leu	1.63	trnL-UAA	UAA	Stop	0	
UUG	Leu	1.11	trnL-CAA	UAG	Stop	0	
CUU	Leu	0.43	trnL-UAG	CAU	His	1.71	trnH-GUG
CUC	Leu	1.2		CAC	His	0.29	
CUA	Leu	0.86		CAA	Gln	0.75	trnQ-UUG
CUG	Leu	0.77		CAG	Gln	1.25	
AUU	Ile	1.06		AAU	Asn	1.52	trnN-GUU
AUC	Ile	1.06		AAC	Asn	0.48	
AUA	Ile	0.87	trnI-CAU	AAA	Lys	1.13	
AUG	Met	1	trnM-CAU	AAG	Lys	0.87	
GUU	Val	1.22	trnV-GAC	GAU	Asp	1.26	trnD-GUC
GUC	Val	0.87		GAC	Asp	0.74	
GUA	Val	1.1		GAA	Glu	0.5	trnE-UUC
GUG	Val	0.81		GAG	Glu	1.5	
UCU	Ser	0.75	trnS-GGA	UGU	Cys	1.67	trnC-GCA
UCC	Ser	2.17		UGC	Cys	0.33	
UCA	Ser	0.9		UGA	Stop	0	
UCG	Ser	0.9	trnS-UGA	UGG	Trp	1	trnW-CCA
CCU	Pro	1.37	trnP-UGG	CGU	Arg	0.92	trnR-ACG
CCC	Pro	1.37		CGC	Arg	1.23	trnR-UCU
CCA	Pro	0.84		CGA	Arg	0.92	
CCG	Pro	0.42		CGG	Arg	1.08	
ACU	Thr	1.58		AGA	Arg	0.62	
ACC	Thr	0.97		AGG	Arg	1.23	
ACA	Thr	0.85	trnT-GGU	AGU	Ser	0.52	trnS-GCU
ACG	Thr	0.61	trnT-UGU	AGC	Ser	0.75	
GCU	Ala	1.29	trnA-UGC	GGU	Gly	1	
GCC	Ala	1.16		GGC	Gly	1.11	trnG-GCC
GCA	Ala	0.65		GGA	Gly	0.61	
GCG	Ala	0.9		GGG	Gly	1.28	

Table S5: The predicted RNA editing sites in *P. acacia* chloroplast genome.

Gene	Nucleotide Position	Amino Acid Position	Codon Conversion	Amino Acid Conversion	Score	position
atpA	773	258	TCA=> TTA	S=>L	1	1
	914	305	TCA=> TTA	S=>L	1	1
atpF	92	31	CCA=> CTA	P=>L	0.86	1
atpI	23	8	ACC=> ATC	T=>I	1	1
	35	12	ACC=> ATC	T=>I	1	1
	641	214	TCA=> TTA	S=>L	1	1
ccsA	137	46	ACA=> ATA	T=>I	1	1
matK	334	112	CTT=> TTT	L=>F	0.86	1
	658	220	CAT=> TAT	H=>Y	1	1
	926	309	GCT=> GTT	A=>V	0.86	1
	1144	382	CCA=> TCA	P=>S	0.86	1
	1393	465	CCA=> TCA	P=>S	0.86	1
petB	418	140	CGG=> TGG	R=>W	1	1
	611	204	TCA=> TTA	S=>L	1	1
psbB	305	102	GCA=> GTA	A=>V	0.86	1
rpl2	601	201	CCG=> TCG	P=>S	1	1
rpl20	308	103	TCA=> TTA	S=>L	0.86	1
rpoA	368	123	TCG=> TTG	S=>L	1	1
	751	251	CTT=> TTT	L=>F	0.86	1
rpoB	338	113	TCT=> TTT	S=>F	1	1
	473	158	TCA=> TTA	S=>L	0.86	1
	566	189	TCG=> TTG	S=>L	1	1
	898	300	CTT=> TTT	L =>F	0.86	1
	2342	781	ACA=> ATA	T=>I	1	1
	2788	930	CTT=> TTT	L=>F	0.86	1
rpoC2	800	267	GCC=> GTC	A=>V	0.86	1
	1241	414	GCG=> GTG	A=>V	0.86	1
	2281	761	CGG=> TGG	R=>W	1	1
	2800	934	CCT=> TCT	P=>S	1	1
rps2	248	83	TCA=> TTA	S=>L	1	1
rps8	182	61	TCA=> TTA	S=>L	0.86	1
	334	112	CCG=> TCG	P=>S	1	1
ycf3	233	78	ACA=> ATA	T=>I	1	1

Table S6: The predicted RNA editing sites in *P. curviflorus* chloroplast genome.

Gene	Nucleotide Position	Amino Acid Position	Codon Conversion	Amino Acid Conversion	Score	Position
atpA	773	258	TCA=>TTA	S=>L	1	1
	914	305	TCA=>TTA	S=>L	1	1
	1148	383	TCA=>TTA	S=>L	1	1
atpF	92	31	CCA=>CTA	P=>L	0.86	1
atpI	23	8	ACC=>ATC	T=>I	1	1
	35	12	ACC=>ATC	T=>I	1	1
	641	214	TCA=>TTA	S=>L	1	1
	644	215	GCG=>GTG	A=>V	0.86	1
ccsA	43	15	CTT=>TTT	L=>F	1	1
	137	46	ACA=>ATA	T=>I	1	1
matK	322	108	CTT=>TTT	L=>F	0.86	1
	646	216	CAT=>TAT	H=>Y	1	1
	914	305	GCT=>GTT	A=>V	0.86	1
	1132	378	CCA=>TCA	P=>S	0.86	1
	1381	461	CCA=>TCA	P=>S	0.86	1
petB	418	140	CGG=>TGG	R=>W	1	1
	611	204	TCA =>TTA	S =>L	1	1
psbB	305	102	GCA=>GTA	A=>V	0.86	1
rpl20	308	103	TCA=>TTA	S =>L	0.86	1
rpoA	368	123	TCG=>TTG	S =>L	1	1
rpoB	338	113	TCT=>TTT	S=>F	1	1
	473	158	TCA=>TTA	S=>L	0.86	1
	551	184	TCA=>TTA	S=>L	1	1
	566	189	TCG=>TTG	S=>L	1	1
	898	300	CTT=>TTT	L=>F	0.86	1
	2342	781	ACA=>ATA	T=>I	1	1
	2788	930	CTT=>TTT	L=>F	0.86	1
rpoC1	41	14	TCG=>TTG	S=>L	1	1
	296	99	ACA=>ATA	T=>I	1	1
rpoC2	815	272	GCG=>GTG	A=>V	0.86	1
	1256	419	GCG =>GTG	A=>V	0.86	1
	2296	766	CGG=> TGG	R=>W	1	1
	2815	939	CCT => TCT	P=>S	1	1
	3986	1329	GCG => GTG	A=>V	0.86	1
	4112	1371	TCT=> TTT	S=>F	0.8	1
rps2	248	83	TCA =>TTA	S=>L	1	1
rps8	182	61	TCA =>TTA	S=>L	0.86	1
	334	112	CCG => TCG	P=>S	1	1
rps14	149	50	CCA=>CTA	P=>L	1	1
ycf3	233	78	ACA=>ATA	T=>I	1	1

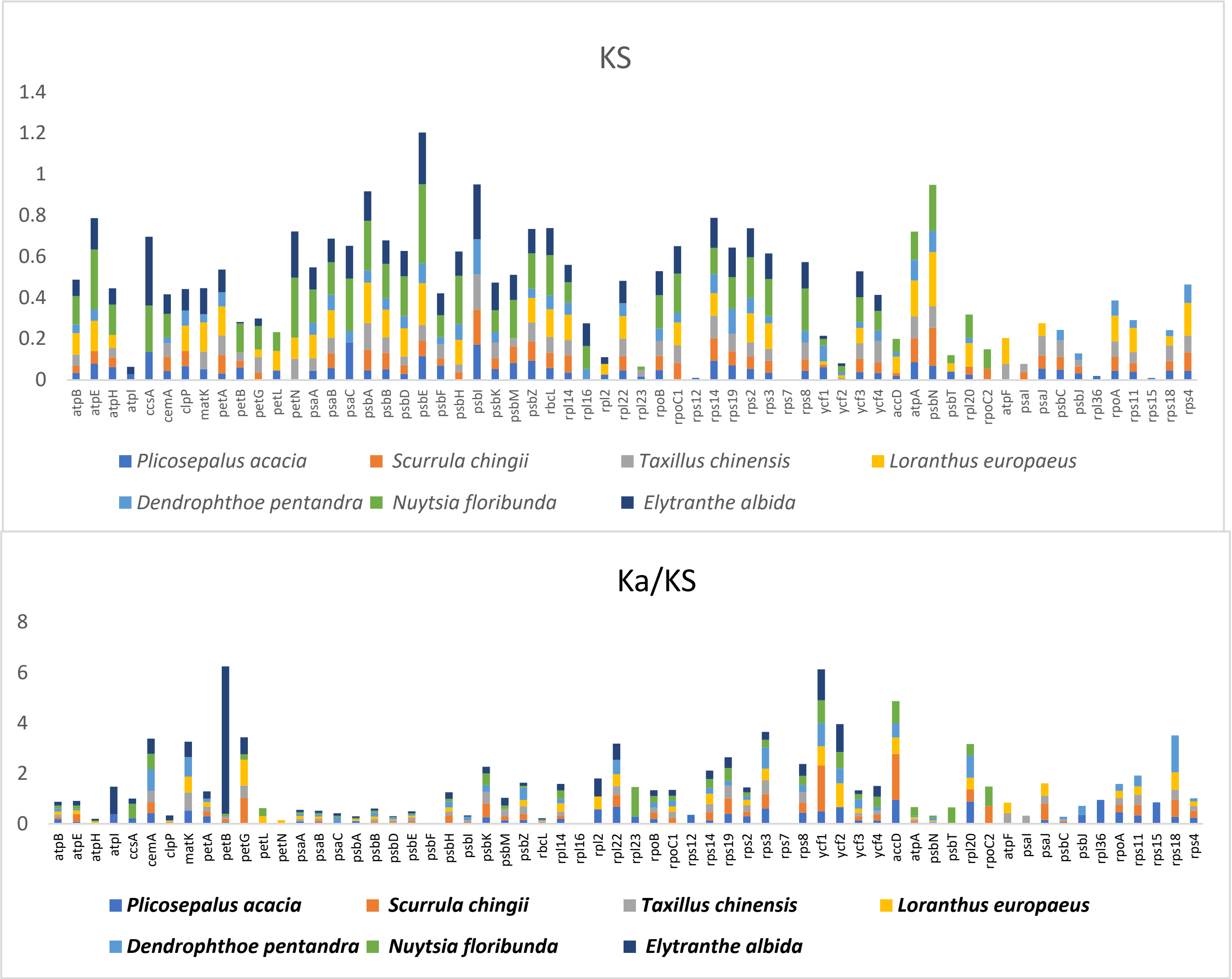

Figure S1: Synonymous (Ks) and Ka/Ks ratio values of 59 protein-coding genes of the *Plicosepalus curviflorus* vs. *Loranthaceae plastomes* (*Plicosepalus acacia*, *Scurrula chingii*, *Taxillus chinensis*, *Loranthus europaeus*, *Dendrophthoe pentandra*, *Nuytsia floribunda* and *Elytranthe*), using the KaKs Calculator 2.0 to detect substitution, selection, and beneficial mutation genes under selective pressure (>1).

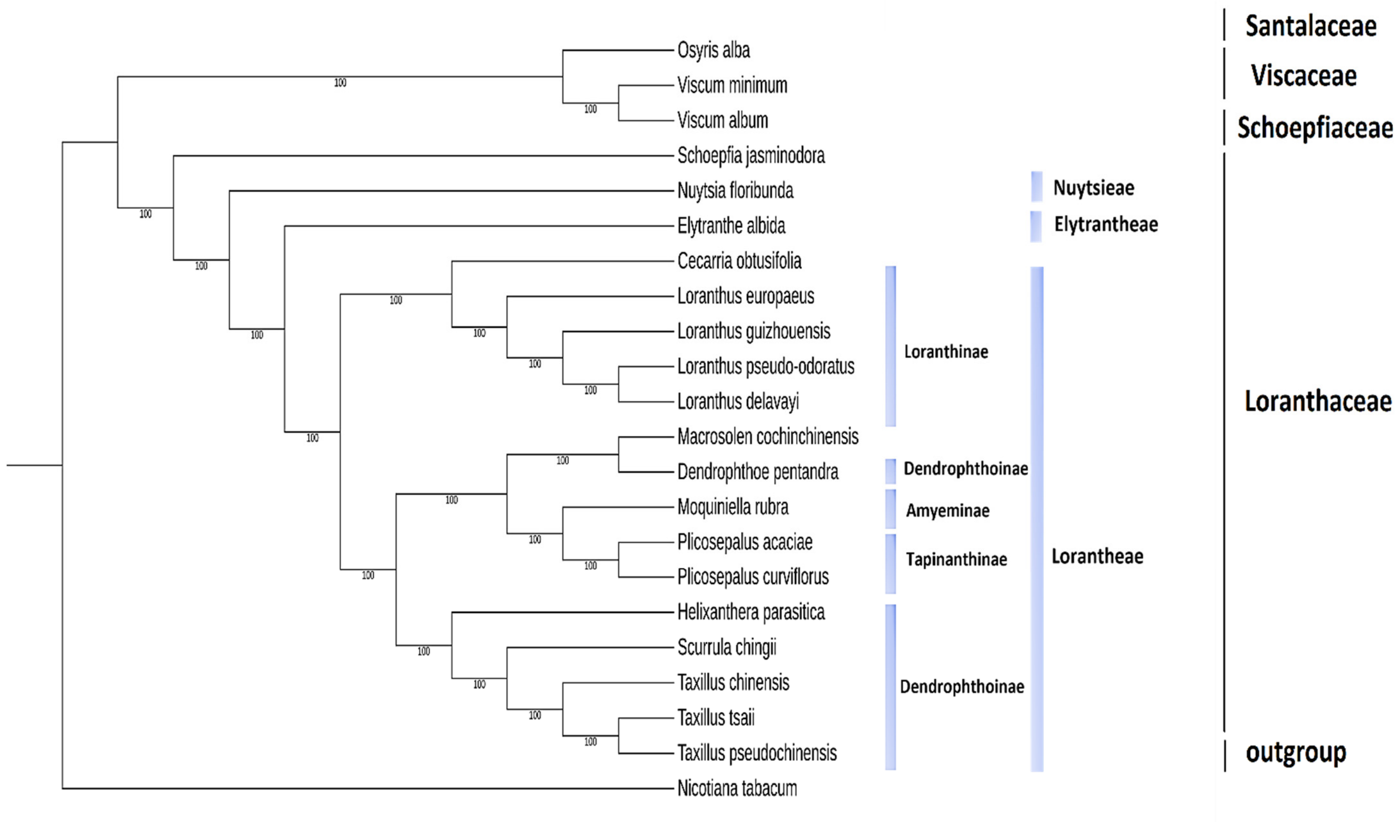

Figure S2: Phylogenetic tree construction inferred from the complete chloroplast genomes of 21 taxa, using Maximum Parsimony (MP) methods. The tree shows the relationships between Brassicales (Cleomaceae, Cappraceae and Brassicaceae). The numbers in the branch nodes represent bootstrap support (BP).
